# Wetting Behavior of Acrylate Hot-Melt Adhesive on Polyester Fabric Substrates and Its Influence on Adhesion Performance

**DOI:** 10.3390/polym18101236

**Published:** 2026-05-19

**Authors:** Haoran Shi, Jun Qian, Yifeng Shi

**Affiliations:** 1 Key Laboratory of Specially Functional Polymeric Materials and Related Technology, Ministry of Education, School of Materials Science and Engineering, East China University of Science and Technology, Shanghai 200237, China; 17664151850@163.com; 2 Shanghai Hongdingfang Technology Co., Ltd., Shanghai 200237, China

**Keywords:** acrylate hot-melt adhesive, temperature, fabric substrate, wetting behavior, apparent surface energy

## Abstract

Acrylate hot-melt adhesives (AHMAs) are widely used in medical dressings, electronic components, and automotive interiors due to their solvent-free nature and high bonding strength. However, their wetting behavior on porous fabric substrates under varying coating temperatures—a critical factor for interfacial adhesion—remains poorly understood. To investigate how coating temperature affects the wetting and adhesion of acrylic hot-melt adhesives on fabric substrates, the apparent surface tension and viscosity of the adhesive (130–160 °C) and the apparent surface energy of the substrate (20–160 °C) were measured. By combining these measurements with contact angle decay curves on steel plates, scanning electron microscopy of cold-brittle cross-sections, and mechanical property tests, the study analysed the effects of temperature on wetting and spreading, penetration depth, and adhesive performance. Results show that with increasing temperature, adhesive surface tension and viscosity decrease, while fluidity improves; substrate surface energy shows no temperature dependence. The penetration depth into the fabric increases from 16 μm to 25 μm, and penetration uniformity gradually improves. However, both peel strength and loop tack continuously decrease with rising temperature, with optimal adhesion at 130 °C. A penetration depth model based on the Washburn equation effectively predicts the penetration behavior. Viscosity accounts for more than 50% of the effect, whilst the wetting factor contributes to a lesser extent. This study provides a theoretical basis for optimizing the coating process of acrylic hot-melt adhesives on fabric substrates.

## 1. Introduction

Acrylate hot-melt adhesives (AHMA), as an important class of environmentally friendly, solvent-free adhesives, feature 100% solid content, zero VOC emissions, fast curing speeds, high bond strength, and excellent aging resistance [1,2]. They comply with increasingly stringent environmental regulations and have become a key development direction in the adhesive industry [3]. In the medical and healthcare sector, products such as adhesive bandages and medical dressings made by laminating AHMA with fabric substrates meet the safety and comfort requirements of medical settings due to their excellent biocompatibility and breathability [4,5]. In the electronics and electrical sectors, their low-temperature rapid curing properties effectively prevent thermal damage to sensitive components caused by high-temperature processing [6,7]. In the automotive industry, their superior mechanical properties and environmental adaptability make them essential supporting materials for applications such as wiring harness tapes and interior bonding [8,9].

Among the numerous applications of acrylic hot-melt adhesives, composite systems with fabric substrates represent one of the most critical product forms [10], and their end-use performance is highly dependent on the quality of the interfacial bond between the adhesive and the substrate [11]. Wetting behavior, as the primary step in interfacial bonding, directly influences the formation and transfer of intermolecular forces, thereby determining key performance indicators such as bond strength and durability [12]. The wetting and spreading ability of the adhesive on the fabric surface not only determines the actual contact area but also affects its penetration depth into the fiber pores, ultimately forming an interfacial mechanical interlocking structure that has a decisive impact on mechanical properties [13]. Therefore, a thorough understanding of its wetting behavior on fabric substrates holds significant theoretical and engineering importance [14].

Temperature is a critical control parameter in the coating process, exerting a dual regulatory effect on the wetting and adhesion performance of the adhesive–substrate system [15]. From the adhesive’s perspective, an increase in temperature intensifies the thermal motion of molecular chains, reduces viscosity, and enhances fluidity, thereby facilitating spreading and penetration [16]; simultaneously, temperature changes alter surface tension and surface energy, thereby affecting the equilibrium of interfacial tension and thermodynamic wetting ability [17]. Research by Taghizadeh et al. [18] confirmed the significant regulatory effect of temperature on the rheological properties of adhesives. From the substrate’s perspective, temperature conduction during the coating process may affect the arrangement of fiber molecular chains and surface molecular density, leading to significant changes in the substrate’s surface energy [19]; Qiu et al. [20] found that the surfaces of fabrics modified with temperature-sensitive materials can undergo reversible hydrophobic/hydrophilic transitions in response to temperature changes, indicating that temperature influences fabric surface properties. Furthermore, the pore structure of fabrics may undergo slight deformations due to temperature changes, thereby affecting the penetration depth of the adhesive and the interfacial contact area [21]. Through these mechanisms, temperature synergistically regulates the wetting and spreading rate, the equilibrium contact angle, and the penetration depth, ultimately determining the quality of the interfacial bond [22]. It should be noted that the surface tension measured under high-temperature dynamic conditions is, in fact, the apparent surface tension. Its value depends not only on intrinsic thermodynamic properties but is also jointly influenced by molecular chain segment motion, surface reorganization kinetics, and the testing time window [15]; its variation results from the combined effects of thermodynamic driving forces and kinetic constraints.

In recent years, scholars both domestically and internationally have conducted extensive research on the application of hot-melt adhesives in fabric lamination [23]. Acrylate hot-melt adhesives have attracted sustained attention due to their excellent aging resistance, transparency, and environmental friendliness [24]. In terms of formulation design, researchers have optimized key properties such as initial tack, peel strength, and holding power by incorporating tackifying resins, crosslinking agents, or functional monomers [25,26]. Regarding rheological properties, the temperature-dependent behavior of viscosity and modulus determines the spreading behavior and penetration depth during the coating process; an appropriate temperature allows the adhesive to achieve good wettability while maintaining sufficient cohesive strength [18]. With respect to fabric substrates, surface energy, fiber morphology, and pore structure significantly influence wettability and penetration behavior [12]. Studies have shown that penetration depth and interfacial bonding state are key factors determining the comprehensive performance of composite fabrics [27]. Increased temperature can significantly reduce viscosity, accelerate spreading, and increase penetration depth; however, excessively high temperatures lead to a decrease in cohesive strength, thereby weakening peel strength [28].

Although significant progress has been made in previous studies, current research on wetting behavior under temperature control still has many shortcomings [29]. First, existing studies have largely focused on single formulations, lacking systematic comparisons of multi-formulation systems; the mechanisms of synergy between temperature and different molecular structures remain unclear [25]. Second, the effect of temperature on wetting behavior results from the combined influence of adhesive and substrate properties; however, existing studies often analyze individual factors in isolation, lacking systematic research on the complete chain of “temperature–adhesive rheological properties–substrate surface properties–wetting behavior” [28]. In particular, there is insufficient research on the temperature response patterns of the pore structure and surface energy of fabric substrates [30]. Furthermore, existing studies are predominantly qualitative in nature, lacking quantitative analysis of the structure–property relationships between wetting behavior and adhesive performance, which makes it difficult to establish a scientific framework for process optimization [18]. Additionally, research on coating processes across a wide temperature range is scarce, and the key factors governing wetting behavior in different temperature ranges remain unclear, leading to process parameter adjustments that rely heavily on empirical experience [24].

Against this background, this study focuses on an acrylic hot-melt adhesive (ODV-2966) applied to a polyester fabric substrate. It systematically investigates the effects of coating temperatures ranging from 130 °C to 160 °C on the adhesive’s apparent surface tension, viscosity, and wetting and spreading behavior, while also examining how temperature influences changes in the substrate’s surface energy. The contact angle decay curves of adhesive droplets on a steel plate surface were recorded in real time using a high-temperature hanging drop apparatus. Scanning electron microscopy (SEM) was employed to observe the cold-fracture cross-sections of composite samples at different temperatures to quantify penetration depth. Combined with peel strength and ring-shaped initial adhesion tests on substrates with varying surface energies—such as steel plate, HDPE plate, and PTFE plate—a structure–property relationship between wetting behavior and adhesive performance was established. Furthermore, a mathematical model of adhesive penetration depth in fabric substrates under temperature-controlled conditions was developed based on the Washburn equation, providing a scientific basis for the formulation design and coating process optimization of high-performance acrylic hot-melt adhesive products [31].

## 2. Materials and Methods

### 2.1. Materials

Acrylate hot-melt adhesive (ODV-2966) and polyester fabric substrate were supplied by Shanghai Hongdingfang Technology Co., Ltd. (Shanghai, China).

The acrylate hot-melt adhesive (ODV-2966) is a commercial-grade acrylate copolymer, a solvent-free thermoplastic pressure-sensitive adhesive with 100% solid content and a density of 1.02 g/cm^3^ at 25 °C.

This polyester fabric substrate is a commercial-grade plain-weave non-woven fabric made from polyethylene terephthalate (PET) fibers.

### 2.2. Equipment

**Figure 1:**
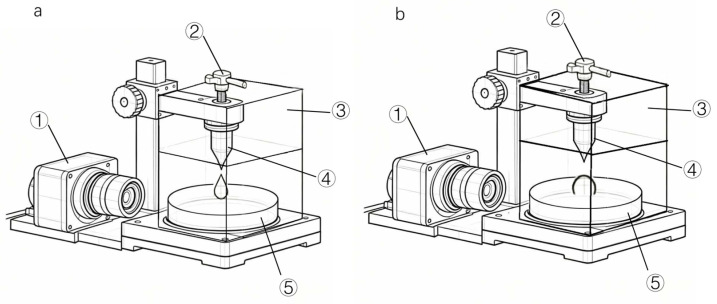
High-temperature testing apparatus: (**a**) Surface tension testing. (**b**) Surface energy testing.

The high-temperature surface tension testing apparatus manufactured by Guangdong Kejian Instrument Co., Ltd., Dongguan, China (as shown in Figure 1) was employed. This apparatus primarily comprises: ① a high-speed camera system; ② an extrusion screw; ③ a temperature-controlled system; ④ a hanging drop system; and ⑤ a temperature-controlled hot stage. Within the temperature-controlled system, the adhesive is maintained in a molten state; the temperature-controlled hot stage allows for precise regulation of the substrate temperature; and the high-speed camera system captures the droplet spreading process in real time. Figure 1a shows the hanging drop method for surface tension measurement using a pendant droplet. Figure 1b shows the sessile drop method for contact angle measurement on a heated stage, with a different sample stage configuration and lighting angle.

### 2.3. Characterization

The apparent surface tension of ODV-2966 at different temperatures was measured using a hanging drop apparatus (Guangdong Kejian Instrument Co., Ltd., Dongguan, China). The apparent contact angle of the substrate at different temperatures was measured using a KJ-625 contact angle measuring instrument (Guangdong Kejian Instrument Co., Ltd., Dongguan, China). N-hexadecane and ethylene glycol were selected as standard liquids, and surface energy was calculated using the Owens-Wendt method. Each sample was tested three times, and the average value was calculated.

The apparent viscosity of ODV-2966 was measured at different temperatures using the RVDV-2H digital viscometer (Shanghai Jing Tian Electronic Instrument Co., Ltd., Shanghai, China) with the shear rate set to 0.8–1.2 s^−1^.

Using a constant-temperature coating system, four temperature gradients of 130 °C, 140 °C, 150 °C, and 160 °C were controlled. With a basis weight of approximately 25 g/m^2^ and at a constant coating speed, ODV-2966 was applied to the fabric substrate to prepare adhesive-substrate composite samples. Composite cross-sections were prepared using the liquid nitrogen embrittlement method. After gold sputtering, the microstructures of the substrate surfaces and cross-sections were observed using Nova Nano SEM 450 Field Emission Scanning Electron Microscope (FEI, Hillsboro Oregon, OR, USA). Pore size and distribution were measured, and quantitative analysis was performed using Image J (1.54 g).

The wetting behavior of ODV-2966 on a steel plate was recorded using the high-temperature hanging drop apparatus (Guangdong Kejian Instrument Co., Ltd., Dongguan, China). After stabilizing ODV-2966 and the steel plate separately in a constant-temperature system for a period of time, ODV-2966 droplets were deposited onto the steel plate using the hanging drop apparatus. Photographs of the droplets were taken every 5 s to record in real time the spreading process of the ODV-2966 on the steel plate surface, and to calculate the spreading rate and the equilibrium apparent contact angle.

In accordance with the GB/T 2792-2014 standard [32,33], peel strength was tested using a KJ-1067 tensile testing machine manufactured by Guangdong Kejian Instrument Co., Ltd., with a test speed of 300 mm/min; A 150 mm × 25 mm adhesive-substrate coating sample strip was adhered to steel plate, HDPE plate, and PTFE plate substrates. The samples were rolled five times with a 2 kg roller, and testing was conducted after 20 min and 24 h, respectively. Each sample was tested three times, and the average value was calculated.

In accordance with the GB/T 31125-2014 standard [34,35], the ring-shaped initial adhesion of the adhesive was tested using the KJ-6031 Ring-Shaped Initial Adhesion Tester manufactured by Guangdong Kejian Instrument Co., Ltd.; A 125 mm × 25 mm adhesive-substrate composite sample strip was adhered to steel, HDPE, and PTFE substrates. After rolling the sample five times with a 2 kg pressure roller, the test was initiated. The instrument performed the tensile test at a speed of 300 mm/min. Each sample was tested three times, and the average value was calculated.

## 3. Results and Discussion

### 3.1. The Effect of Temperature on the Apparent Surface Tension of AHMA

**Figure 2:**
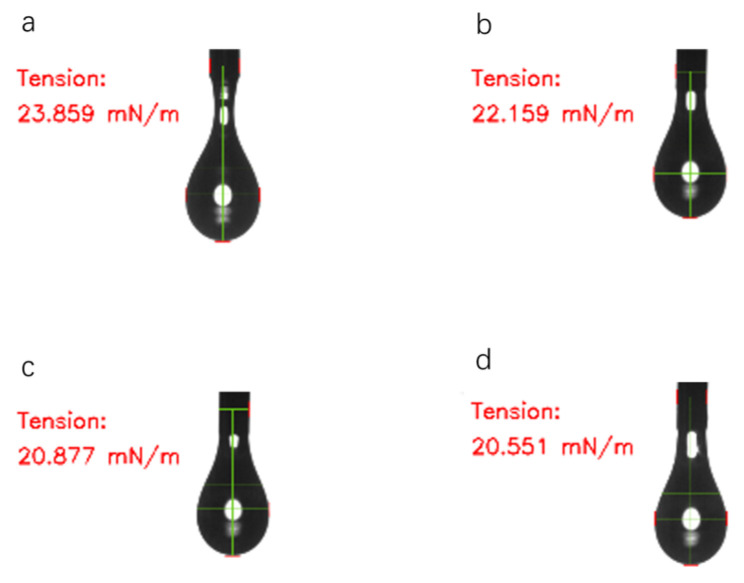
Images of the apparent surface tension of ODV-2966 at 130 °C (**a**), 140 °C (**b**), 150 °C (**c**), and 160 °C (**d**).

**Figure 3:**
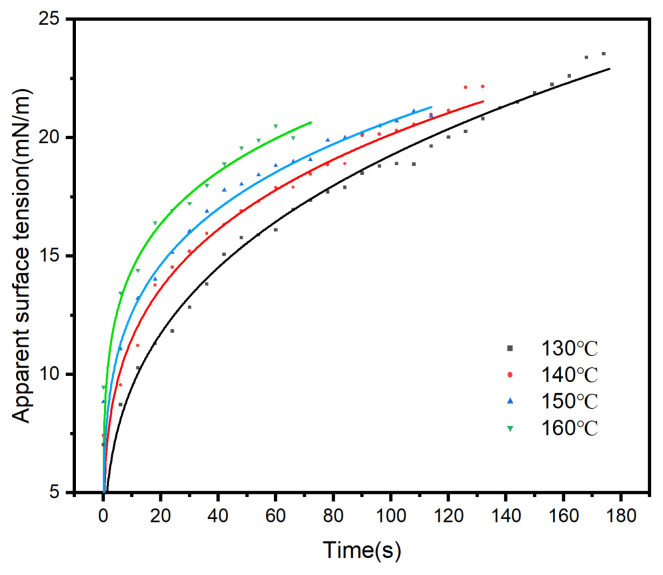
Apparent surface tension of ODV-2966 at different temperatures. (Black:130 °C, Red:140 °C, Blue:150 °C, Green:160 °C).

Using a high-temperature hanging drop apparatus with a hanging drop needle having an outer diameter of 0.8 mm (inner diameter of 0.5 mm), hot-melt adhesive droplets were extruded at a constant rate. The surface tension values prior to the final drop are shown in Figure 2. A photograph was taken every 6 s during the extrusion process. The software automatically calculated and recorded the apparent surface tension values of the adhesive droplets, as shown in Figure 3. The selected test temperature range (130–160 °C) corresponds to the typical coating window for acrylate hot-melt adhesives in industrial applications such as medical tape lamination and electronics component assembly [25,28]. Four specific temperatures (130, 140, 150, and 160 °C) were chosen to evenly cover this range with a 10 °C interval, allowing systematic observation of temperature-dependent trends in viscosity, surface tension, and wetting behavior.

Figure 3 shows the time-dependent changes in the apparent surface tension of ODV-2966 at different temperatures.

**Figure 4:**
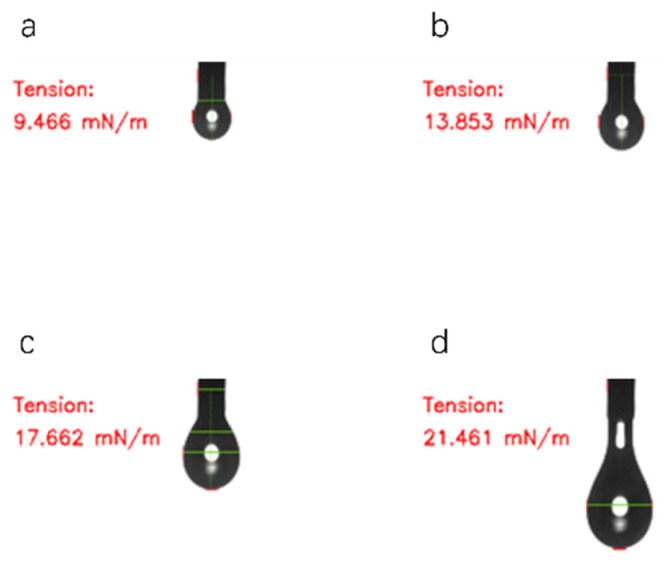
The apparent surface tension of ODV-2966 at 160 °C after 6 s (**a**), 24 s (**b**), 48 s (**c**), 60 s (**d**).

Figure 4 shows the change in the apparent surface tension of ODV-2966 over time at 160 °C (photograph taken during the droplet formation process).

As shown in Figure 3, within the temperature range of 130–160 °C, the apparent surface tension of ODV-2966 exhibits a continuous downward trend with increasing temperature over the same time period. At the same time, the apparent surface tension at each temperature gradually increases as the test duration extends, reflecting the dynamic behavior of molecular chains aligning along the orientation direction and surface-layer molecules tending toward order during the droplet stretching process. From the perspective of temperature response mechanisms, the decrease in apparent surface tension with increasing temperature is consistent with classical thermodynamic principles: heating intensifies the thermal motion of molecular segments, weakens intermolecular interactions, and reduces the tendency of surface-layer molecules to contract inward; meanwhile, high temperatures further enhance the mobility of molecular chains, causing the apparent surface tension to become more stable under high-temperature conditions. This dual dependence on temperature and time indicates that the low apparent surface tension at high temperatures provides favorable thermodynamic conditions for the wetting and spreading of the adhesive on the substrate surface.

### 3.2. The Effect of Temperature on the Rheological Properties of AHMA

**Figure 5:**
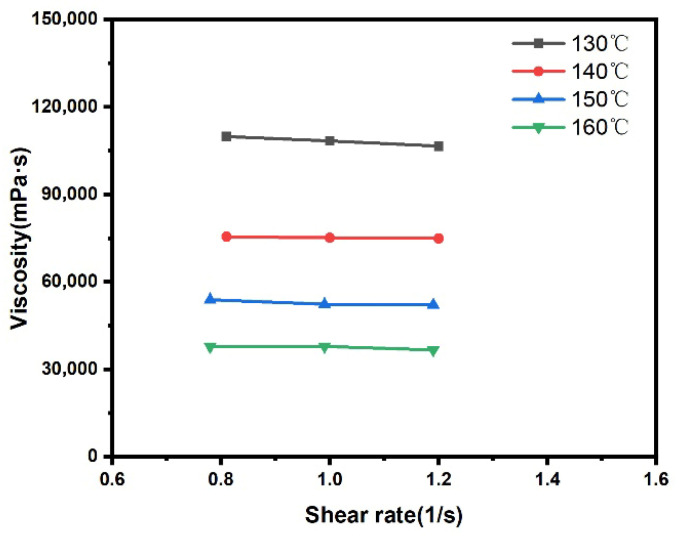
Viscosity of ODV-2966 at different temperatures and shear rates.

This section examines the apparent viscosity of ODV-2966 at different temperatures; tests were conducted at three shear rates for each temperature, with the results shown in Figure 5.

**Figure 6:**
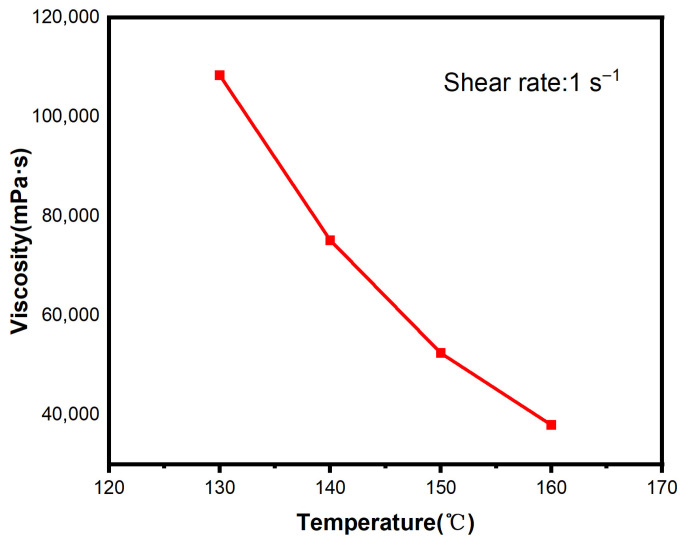
Viscosity of ODV-2966 at different temperatures.

As can be seen from Figure 5, the apparent viscosity of ODV-2966 at a given temperature remains essentially constant as the shear rate increases. Therefore, the viscosity value at a shear rate of 1 s^−1^ was selected for subsequent calculations; the results are shown in Figure 6.

Within the temperature range of 130 °C to 160 °C, the viscosity of ODV-2966 exhibits a significant downward trend as the temperature rises. When the temperature increases from 130 °C to 160 °C, the viscosity decreases from 1.1 × 10^5^ mPa·s to 3.8 × 10^4^ mPa·s, representing a 65% reduction. This indicates that the temperature increase effectively reduces the flow resistance of the melt, significantly improving its flowability. This change is primarily attributed to the increased thermal motion of the polymer molecular chains: as the temperature rises, the molecular segments acquire more thermal energy, intermolecular interactions weaken, the density of the entangled network decreases, and the mobility of the segments increases, thereby reducing the flow resistance of the melt. The temperature dependence of viscosity has a significant impact on the hot-melt adhesive coating process: at lower temperatures, viscosity is higher, limiting the adhesive’s flowability and making it difficult to spread rapidly over the substrate surface and penetrate into fiber pores; as the temperature rises, viscosity decreases significantly, enhancing the adhesive’s flowability, which facilitates its wetting and spreading over the substrate surface as well as capillary penetration into the fiber bundles. By controlling the coating temperature, the flow behavior of the adhesive can be effectively optimized, thereby improving the quality of the interface bond with the fabric substrate.

### 3.3. The Effect of Temperature on the Apparent Surface Energy of Substrate

The surface of polyester fabric substrates exhibits a typical interwoven fiber structure and a porous morphology. To investigate the effect of temperature on the apparent surface energy of the substrate, in this series of experiments, n-hexadecane was used in place of water as the nonpolar test liquid, which helps mitigate, to some extent, the interference caused by the evaporation of the test liquid at high temperatures on contact angle measurements. A 2 μL droplet was placed in contact with the sample surface, and the apparent contact angles were measured at different temperatures. The contact angle values were then substituted into the Owens-Wendt equation to calculate the apparent surface energy of the substrate, as shown in Table 1.

As shown in Table 1, within the temperature range of 20–160 °C, the apparent contact angles of polyester fabrics for n-hexadecane and ethylene glycol did not exhibit any obvious monotonic trend. The contact angle for n-hexadecane fluctuated slightly between 125° and 132°, while that for ethylene glycol remained within the range of 98–100°. The apparent surface energy calculated using the Owens-Wendt method ranged from 11.0 to 13.5 mN/m. It can be concluded that within the temperature range of 20–160 °C, the apparent surface energy of polyester fabrics does not exhibit significant temperature dependence; that is, the effect of temperature on the apparent surface energy of this substrate can be neglected. This conclusion provides a prerequisite for the stability of the substrate’s surface properties in subsequent studies of the wetting and penetration behavior of adhesives on fabric substrates at coating temperatures (130–160 °C).

### 3.4. Temperature Dependence of the Adhesive Wetting Process

**Figure 7:**
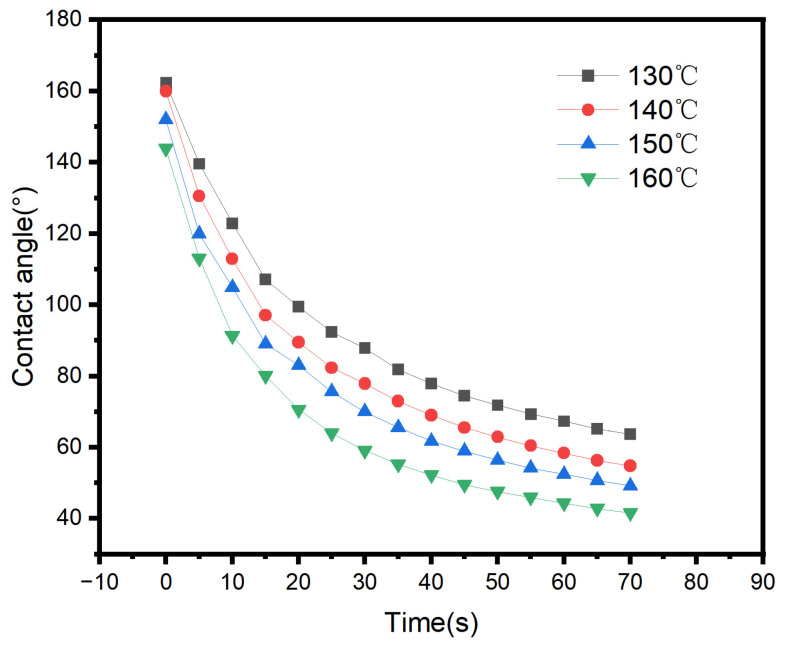
Changes in the apparent contact angle of ODV-2966 on a steel plate surface over time at different temperatures.

Due to the significant opacity and surface roughness of the fabric substrate, it is difficult to directly observe the dynamic spreading process of adhesive droplets using optical methods. The steel plate used in peel strength testing serves as a high-energy, uniform surface with a surface roughness requirement of (50 ± 25) nm. Compared to fabric substrates, it significantly reduces the interference of fiber capillary effects and surface roughness on spreading kinetics, thereby providing a purer reflection of how the adhesive’s own rheological properties and surface tension influence wetting behavior. Therefore, this study employs the steel plate used in peel strength testing as a model substrate. By recording the contact angle decay curves of the adhesive on the steel plate surface in real time at different temperatures, as shown in Figure 7, the study indirectly evaluates its intrinsic spreading behavior on fabric substrates.

**Figure 8:**
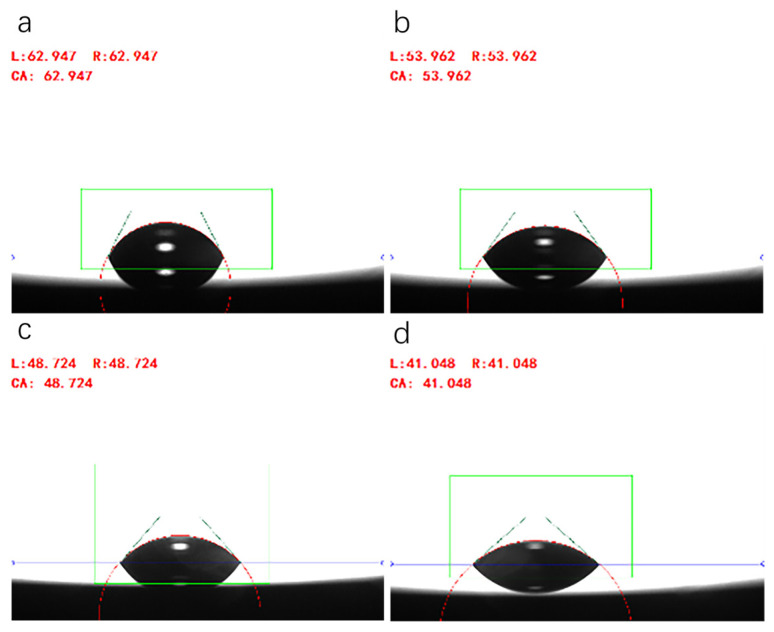
The apparent contact angle of ODV-2966 on the steel plate. (**a**) 130 °C, (**b**) 140 °C, (**c**) 150 °C, (**d**) 160 °C.

Figure 8 shows the apparent contact angle of ODV-2966 on a steel plate surface at different temperatures, with t = 70 s.

As shown in Figure 7, within the temperature range of 130–160 °C, the rate at which the apparent contact angle of the adhesive on the steel plate surface decreases over time accelerates significantly with rising temperature, and the equilibrium contact angle continues to decrease, indicating that high temperatures significantly improve the adhesive’s spreading ability. This phenomenon is primarily attributed to the fact that increased temperature causes a substantial decrease in the adhesive’s viscosity and intensifies the thermal motion of molecular chains, thereby accelerating the spreading kinetics of the droplet on the solid surface; simultaneously, the surface tension of the adhesive decreases at high temperatures, further reducing the interfacial tension between the solid and liquid phases, which facilitates the reduction in the contact angle. Although there are differences in the surface properties of steel plates and fabric substrates, the wetting behavior of the adhesive on the steel plate directly reflects the temperature-dependent variation in its intrinsic spreading ability. Therefore, it can be inferred that at the same coating temperature, the adhesive will similarly exhibit an enhanced wetting and spreading trend on the fabric substrate as the temperature rises, providing favorable conditions for achieving deeper capillary penetration into the fabric in subsequent processes.

### 3.5. The Effect of Temperature on Penetration Depth

**Figure 9:**
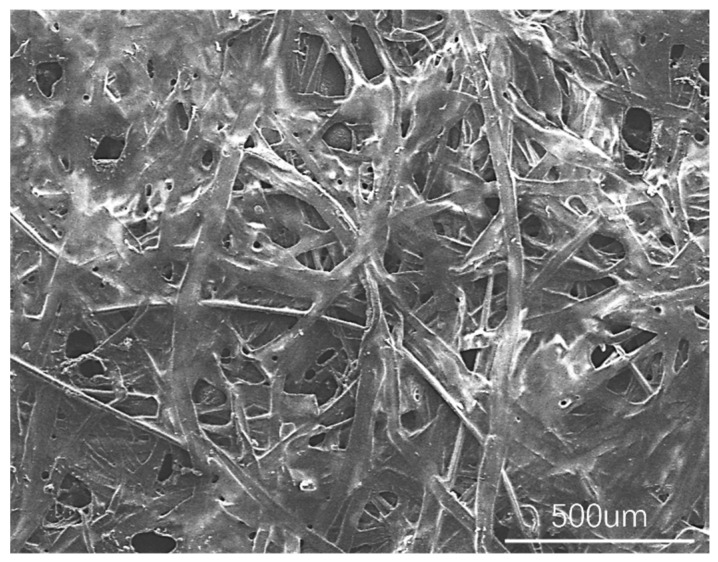
Microstructure of the substrate surface.

Figure 9 shows SEM images of the surface of the polyester fabric substrate at different magnifications at room temperature. As shown in the figure, the fabric substrate consists of a typical interwoven fiber structure, with the fibers oriented isotropically and having diameters ranging from 5 to 20 μm. The surface of individual fibers is relatively smooth, and some fibers exhibit fiber splitting and microfibrillation. The inter-fiber bonding points are relatively dense, forming a large number of inter-fiber pores. The pore sizes are primarily concentrated in the 5–40 μm range, exhibiting a rich structure of open pores and capillary channels. The substrate exhibits good overall uniformity and high surface flatness, with a small number of fine fibers visible locally filling the pores. This surface morphology provides a favorable structural foundation for the wetting, spreading, and capillary penetration of the adhesive.

**Figure 10:**
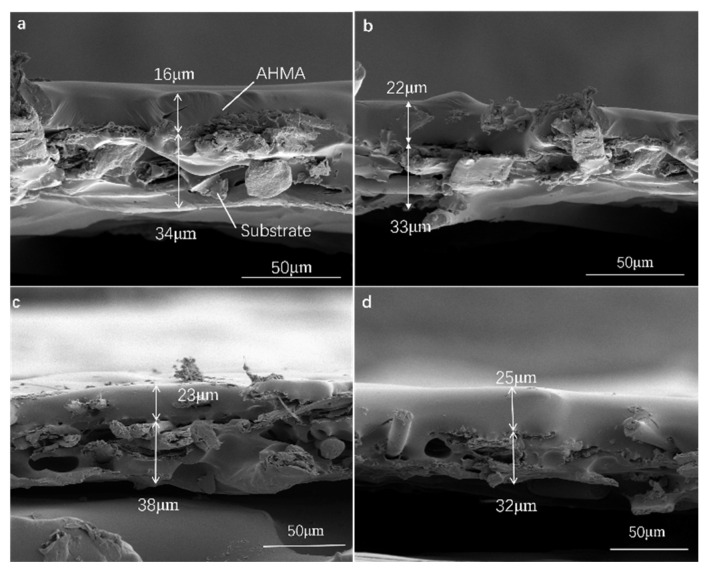
Morphology of the permeable cross-section at coating temperatures of 130 °C (**a**), 140 °C (**b**), 150 °C (**c**), and 160 °C (**d**).

**Figure 11:**
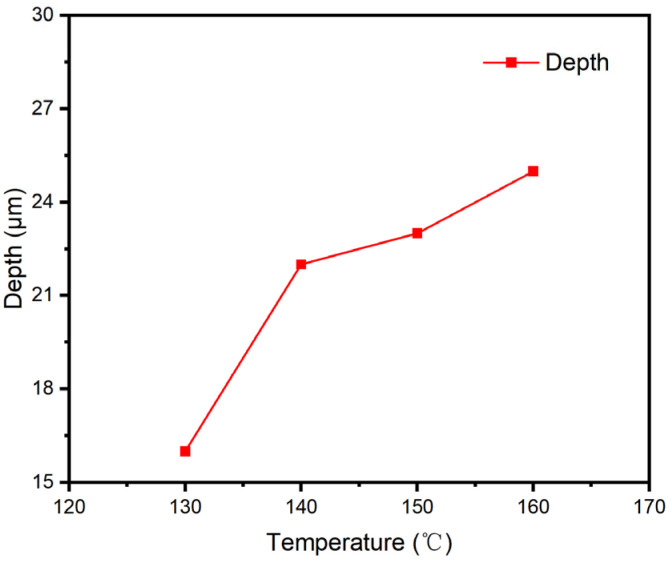
Penetration depth of ODV-2966 into the substrate at different temperatures.

On an industrial coating machine, coating samples of ODV-2966 adhesive on a polyester fabric substrate were obtained by controlling the temperature of the hot-melt adhesive and the backing roller at the die opening. Figure 10 shows SEM images of the penetration cross-sections of ODV-2966 within the substrate at 130 °C (a), 140 °C (b), 150 °C (c), and 160 °C (d). Plotting the penetration depth of the adhesive layer against temperature yields Figure 11.

As shown in Figure 11, within the coating temperature range of 130 °C to 160 °C, the penetration depth of the adhesive into the fabric substrate increases significantly with rising temperature, from 16 μm at 130 °C to 25 μm at 160 °C. This change is not due to a temperature-induced response in the substrate’s pore structure, but rather results from the synergistic evolution of the adhesive’s physical properties with temperature. On the one hand, viscosity decreases sharply with rising temperature, significantly reducing flow resistance, which helps the adhesive overcome the capillary resistance of the fiber network and penetrate into deeper channels. On the other hand, the apparent surface tension of the adhesive decreases with rising temperature, while the apparent surface energy of the substrate remains stable within the 130–160 °C range; the increase in the wetting factor further enhances the capillary driving force. Therefore, the temperature dependence of penetration depth is essentially a viscosity-dominated, wetting-synergistic regulatory mechanism: at low temperatures, high viscosity restricts flow, and the adhesive primarily fills the inter-fiber pores in the surface layer; as the temperature rises, the decrease in viscosity and improved wetting jointly promote the adhesive’s stepwise penetration into the middle and deep layers of the fiber bundles, forming a continuous, dense penetration layer by 160 °C.

### 3.6. The Effect of Temperature on the Mechanical Properties of Adhesive-Substrate Composite Systems

**Figure 12:**
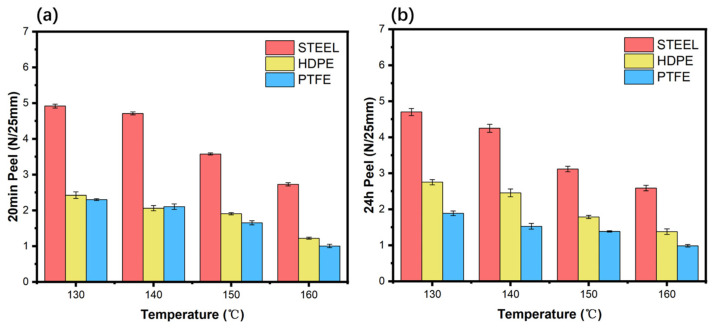
20 min peel strength (**a**) and 24 h peel strength (**b**) of ODV-2966 on different substrates at various coating temperatures. All failure modes are adhesive failures.

As shown in Figure 12, within the temperature range of 130 °C to 160 °C, the peel strength on all substrates exhibits a continuous downward trend as the temperature increases. Although increased temperature significantly reduced the adhesive viscosity (by 65%), decreased the contact angle, and increased the penetration depth, excessively high coating temperatures also caused excessive molecular chain motion in the adhesive, potentially partially disrupting the cross-linking density or entangled network, resulting in a reduction in the effective thickness of the adhesive layer, which in turn leads to a significant decrease in cohesive strength. Direct and accurate measurement of the residual adhesive layer thickness by SEM is difficult because the adhesive fills the internal pores of the fabric, obscuring a clear boundary between the surface layer and the penetrated region. Consequently, the reduction in effective thickness is inferred from the penetration depth trend, not from direct thickness measurements. Peel strength is a comprehensive reflection of interfacial bonding force and cohesive strength within the adhesive layer; when the loss of cohesive strength exceeds the gain in interfacial wetting, the overall peel performance declines. The results of this experiment indicate that within the range of 130 °C to 160 °C, the weakening of cohesive strength plays a dominant role; therefore, 130 °C is the optimal coating temperature for this adhesive system, at which the peel strength is highest.

**Figure 13:**
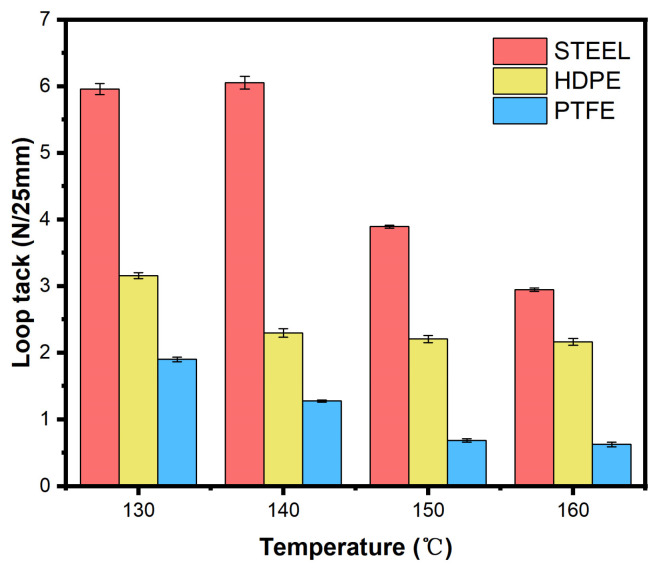
Initial ring adhesion of ODV-2966 on different substrates at various temperatures. All failure modes are adhesive failures.

Figure 13 shows the ring-shaped initial adhesion of ODV-2966 at different coating temperatures. Overall, ring-shaped initial adhesion decreases as temperature increases. For steel plates, initial adhesion is relatively high at 130–140 °C but decreases significantly after 150 °C; HDPE and PTFE, on the other hand, exhibit a continuous downward trend. At low temperatures, higher viscosity provides greater resistance to deformation, which is beneficial for instantaneous adhesion; at high temperatures, reduced viscosity and weakened cohesive strength lead to a decrease in initial ring adhesion. Combined with the penetration depth results, high temperatures promote the penetration of the adhesive into the substrate, but the loss of cohesive strength has a more significant negative impact on initial adhesion. Therefore, lower coating temperatures (130–140 °C) are more conducive to achieving higher initial ring adhesion, and this advantage is particularly pronounced for low-energy surfaces such as PTFE.

### 3.7. Validation of Mathematical Models of Wetting Behavior

According to the classical capillary permeation theory (Washburn equation [36]), the permeation behavior of a liquid in a porous medium can be described by the following equation:

(1) $$\frac{dL}{dt}=\frac{R\gamma_{L}cos\theta }{4\eta L}$$

In the equation, *L* represents the penetration depth, *t* represents time, *R* represents the capillary equivalent radius, $\gamma_{L}$ represents the surface tension of the adhesive, *θ* represents the apparent contact angle, and η represents the viscosity of the adhesive.

Integrating the above expression yields the functional relationship between penetration depth and time:

(2) $$L^{2}=\frac{R\gamma_{L}cos\theta }{2\eta L}\cdot t$$

For porous systems such as fabric substrates, the pore structure does not consist of ideal parallel capillaries; therefore, a structural factor *ϕ* (which accounts for factors such as pore tortuosity and pore size distribution) must be introduced to correct for this. Additionally, given that the adhesive penetration time is fixed in this study (the time window between coating and curing), Equation (2) can be rewritten as

(3) $$D=K\cdot \sqrt{\frac{\gamma_{L}cos\theta }{\eta }}$$

Here, *D* represents the macroscopic penetration depth (μm); *K* represents the comprehensive structural constant (μm $\cdot {Pa}^{\frac{1}{2}}\cdot s^{\frac{1}{2}}\cdot {mN}^{\frac{1}{2}}$), which incorporates the effects of substrate pore characteristics (*R*, *ϕ*) and time; $\gamma_{L}$ is the surface tension of the adhesive (mN/m); θ is the apparent contact angle of the adhesive on the substrate surface (°); η is the viscosity of the adhesive (Pa·s).

When using the Washburn equation to establish a penetration depth model, the effective contact angle between the adhesive and the pore walls of the substrate must be known. Since the substrate surface is rough and porous, making direct measurement difficult, this study employs the Neumann equation of state [37] to estimate the contact angle. This equation, based on interfacial thermodynamics, establishes an empirical relationship between the solid–liquid contact angle and the solid surface energy and liquid surface tension, as shown in Equation (4). It does not require the polar and dispersive components of the solid surface energy and is suitable for low-energy surface systems. By substituting the apparent surface tension of the adhesive, $\gamma_{L}$, and the apparent surface energy of the substrate, $\gamma_{S}$, into this equation, the effective contact angle, θ, at various temperatures can be estimated and subsequently substituted into Equation (3) for model fitting.

(4) $$cos\theta =-1+2\sqrt{\frac{\gamma_{S}}{\gamma_{L}}}exp[-0.000125{\left(\gamma_{L}-\gamma_{S}\right)}^{2}]$$

Let $\gamma_{S}$ = 13.0 mN/m, and let $\gamma_{L}$ be taken from Figure 2. Calculate the values of *cosθ* and *θ* at each temperature; the results are shown in Table 2.

Based on experimental data obtained at coating temperatures ranging from 130 °C to 160 °C, this study determined the physical parameters in the model, as shown in Table 3. The adhesive viscosity (η) was derived from viscosity test results at different temperatures; the viscosity value corresponding to a shear rate of 1.0 $s^{-1}$ was selected, and the units were converted to Pa·s. The surface tension of the adhesive ($\gamma_{L}$) was obtained from surface tension test results within the range of 130 °C to 160 °C. The apparent contact angle of the adhesive on the substrate surface was derived from the effective contact angle calculated using the Neumann equation of state. The penetration depth (*D*) was obtained from SEM observations of the cold-brittle cross-section.

Substituting the above parameters into Equation (3), we calculated the values of $\sqrt{\gamma_{L}cos\theta /\eta }$ at various temperatures and performed a linear regression with the experimental penetration depths, yielding a structural constant K = 50.5 μm${\cdot Pa}^{\frac{1}{2}}\cdot s^{\frac{1}{2}}\cdot {mN}^{\frac{1}{2}}$ and a coefficient of determination R^2^ = 0.98.

This yields the mathematical model for the penetration depth of hot-melt adhesive in fabric substrates:

(5) $$D=50.5\sqrt{\frac{\gamma_{L}cos\theta }{\eta }}\;(R^{2}=0.98)$$

In the equation, *D* represents the penetration depth (μm), $\gamma_{L}$ represents the surface tension of the adhesive (mN/m), and θ represents the apparent contact angle of the adhesive on the substrate surface (°); η is the viscosity of the adhesive (Pa·s), and the constant 50.5 comprehensively reflects the pore structure characteristics of the fabric substrate (equivalent radius, tortuosity, porosity) as well as the influence of the coating process time window.

**Figure 14:**
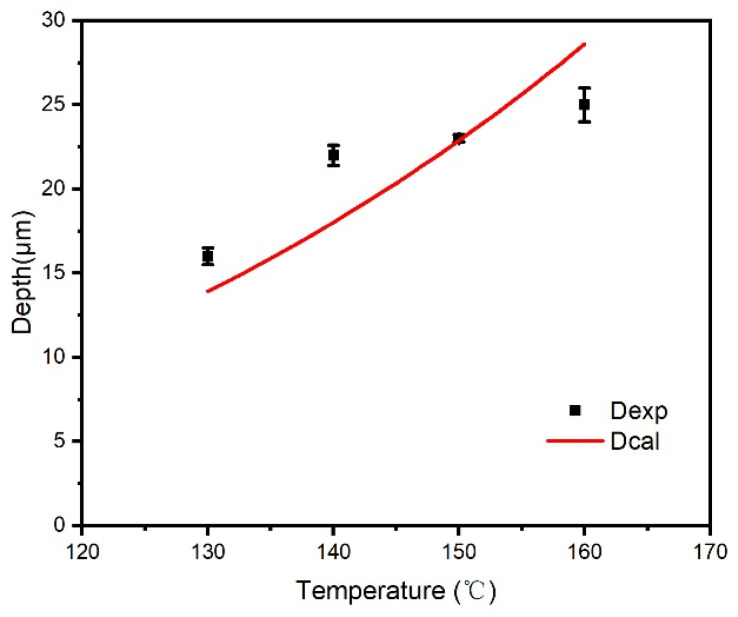
Model-predicted versus experimental values for penetration depth.

Table 4 and Figure 14 show that within the temperature range of 130 °C to 160 °C, the trends in the measured and modelled permeation depths are in close agreement, indicating a high degree of accuracy in the fit. This suggests that penetration depth is primarily determined by the combined effects of the adhesive’s viscosity and wettability, with viscosity having a more significant influence: as the temperature rises from 130 °C to 160 °C, the viscosity decreases by approximately 65%, contributing to an increase in penetration depth of more than 50%; meanwhile, the change in the wetting factor ($\gamma_{L}cos\theta$) makes a relatively minor contribution. The model validated the regulatory mechanism by which temperature promotes the penetration depth of the adhesive into the fabric substrate through the synergistic effects of reducing viscosity and fine-tuning wettability.

## 4. Conclusions

(1) The experimental results indicate that, although high temperatures improve the adhesive’s wetting, spreading and penetration depth, the reduction in the thickness of the remaining hot-melt adhesive layer will result in a decline in mechanical properties. Therefore, in this study, coating temperature should not exceed 140 °C for this adhesive–substrate system to avoid performance loss.
(2) First quantitative model of temperature-dependent penetration depth based on Washburn equation with a structural constant K = 50.5; identification of viscosity as the dominant factor (>50%) over wetting; a relationship between penetration depth and cohesive strength has been identified, providing a theoretical basis for the industrial optimisation of coating process conditions. A limitation of the model is that the effective contact angle was estimated using the Neumann equation rather than directly measured on fabric pore walls—direct measurement is not feasible with conventional methods. This simplification may introduce uncertainty into absolute penetration depth predictions.
(3) In future, provided experimental conditions permit, high-speed X-ray micro-computed tomography (X-ray micro-CT) can be employed to observe capillary penetration in real time and in situ, which will aid in a deeper understanding of the dynamic changes occurring during the wetting process.

## Figures and Tables

**Table 1:** Apparent contact angle and apparent surface energy of substrates at different temperatures.

Substrate	Temperature /°C	Contact Angle of n-Hexadecane /°	Contact Angle of Ethylene Glycol /°	Apparent Surface Energy /mN/m
Polyester fabric	20	125.3	98.7	12.5
Polyester fabric	40	127.5	98.1	13.0
Polyester fabric	60	127.9	99.6	11.7
Polyester fabric	80	129.4	99.2	12.8
Polyester fabric	100	132.2	100.6	12.5
Polyester fabric	120	131.7	98.3	13.5
Polyester fabric	140	130.1	101.2	11.0
Polyester fabric	160	129.5	97.4	13.2

**Table 2:** Apparent contact angle of ODV-2966 on the substrate at different temperatures.

Temperature /°C	$\gamma_{L}$ /mN/m	$\gamma_{S}$ /mN/m	cosθ	θ /°
130	23.9	13.0	0.33	70.7
140	22.2	13.0	0.43	64.6
150	20.9	13.0	0.55	56.7
160	20.6	13.0	0.58	55.2

**Table 3:** Experimental data at different temperatures.

Substrate	Temperature /°C	Viscosity /mPa·s	Apparent Surface Tension /mN/m	Surface Contact Angle /°	Penetration Depth /μm
ODV-2966	130	108,387	23.9	70.7	16.0
ODV-2966	140	75,167	22.2	64.6	22.0
ODV-2966	150	52,429	20.9	56.7	23.0
ODV-2966	160	37,919	20.6	55.2	25.0

**Table 4:** Model-predicted versus experimental values for penetration depth.

Temperature/°C	$D_{exp}$/μm	$D_{cal}$
130	16.0 ± 0.5	13.6
140	22.0 ± 0.6	18.0
150	23.0 ± 0.2	23.6
160	25.0 ± 1.0	28.1

## Data Availability

The original contributions presented in this study are included in this article. Further inquiries should be directed to the corresponding author.
